# Research on mechanical characteristics and key technology of integral intelligent lifting construction of large-span heavy steel box girder

**DOI:** 10.1371/journal.pone.0326918

**Published:** 2025-06-26

**Authors:** Xiaofeng Liu, Mingliang Li, Zhi Geng, Shuhong Zhu, Wenjie Li, Bin Liang

**Affiliations:** 1 The First Construction Co., Ltd. of China Railway Construction 15th Group, Xi’an, Shaanxi, China; 2 School of Civil Engineering and Architecture, Henan University of Science and Technology, Luoyang, Henan, China; Tecnológico de Monterrey, MEXICO

## Abstract

This paper aims to address the problems of aerial orientation control and safety in sudden extreme working conditions during the integral intelligent lifting of large-span heavy steel box girder. Based on the project of Xiaotun Bridge of Fuyi Expressway, a new integral lifting system and control method were proposed. To prevent extreme conditions during the lifting process, the mechanical properties of steel box girders in both synchronous and asynchronous integral lifting states were investigated using the finite element method. Moreover, the mechanical properties of the steel box girders during the lifting process were analyzed through on-site monitoring. The results show that an alignment device has been added to the existing synchronous hydraulic lifting system, which achieves precise control of the aerial orientation and stress of each component of the steel box girder through informal lifting, micromotion lifting and other methods. In the synchronous lifting condition, the failure of the lifting point will cause the redistribution of internal forces in the lifting sling, thereby endangering the lifting lugs, but the stress and deformation of the steel box girder change relatively little. In the asynchronized lifting condition, the use of double lifting points may potentially result in overturning and torsion of the girder. However, under the asynchronous lifting within the displacement error limits, the overall mechanical performance of the steel box girder meets the codes. The on-site monitoring results correlate closely with the simulation results for the normal lifting conditions of the steel box girder. The maximum stress and vertical displacement of the steel box girder are approximately 74 MPa and 70 mm, respectively, indicating that the overall girder structure is safe and reliable throughout the entire lifting process.

## 1 Introduction

With the acceleration of urbanization, there has been a significant increase in the demand for infrastructure, including public buildings and bridges. Steel structures are preferred for large-span space and bridge constructions due to their high strength, lightweight, and excellent seismic performance, etc. However, it is a challenge to erect large span steel structures accurately in the intended location., particularly in complex construction environments. The integral lifting of large sections is commonly employed in the construction of large-span steel structures and steel box girder bridges, owing to the flexibility of the lifting equipment, as well as its safety, efficiency, and suitability for intricate environments.

Application of integral lifting technology in large-span space steel structures includes connecting corridors [1], roofs [2–3], and cold boxes [4]. Concurrently, numerous researchers have explored the management of mechanical properties during the lifting operations of these expansive structures. Zhao [5] investigated the impact of thermal loads on the support reaction forces of complex steel frameworks, and analyzed the variations in lifting height differences at various lifting points. Zhao [6] proposed a real -coded genetic algorithm to optimally analyze the challenges encountered during the erection of complex large steel structures, including the optimal layout of lifting points, temporary supports, and unloading sequences. Tian [7] conducted simulations and analyses of both synchronous and asynchronous integral lifting processes for the steel roofs of Eastern Airlines hangars, and proposed a precise and user-friendly method for simulating lifting operations during the construction of large-span space steel structures. Yang [8] proposed a time-varying mechanical analysis technique for the synchronous and asynchronous integral lifting of large-span space steel structures, and investigate the adverse effects arising from displacement discrepancies between lifting points during asynchronous integral lifting through numerical simulation. Ruan [9] conducted a comprehensive simulation of the lifting process of the steel connecting corridor subjected to wind loads, examined the stress, displacement, and stability of the structure. Zhu [10] monitored the mechanical behavior of the steel structure at the world’s longest large-span converter station, and establish a real-time structural monitoring system for the multi-point lifting process of large-span spatial steel structures.

In the construction of steel box girder bridges the integral lifting technology has been widely utilized [11–13]. The construction of large-section girders through integral lifting involves several stages, including fabrication, transportation, lifting, and welding connections. Throughout these stages, the geometrical configuration of the girders undergoes multiple transformations, which can affect the precision of bridge alignment. Numerous researchers have investigated key construction techniques associated with the integral lifting of large-section steel box girders, considering various project overview [14–16]. Zhang [17] conducted a comparative analysis of the selection of lifting equipment for steel box girders, taking into account the complexities of the construction environment on-site, optimized the positioning of lifting points, and evaluated the safety of the integral lifting process for large-section steel box girders using numerical simulation. Wen [18] provided a comprehensive overview of safety measures related to steel box girder lifting, analyzed the stress and displacement of the girders under varying parameters using numerical simulation, and subsequently proposed a monitoring scheme for the lifting process. Song [19] conducted a comprehensive comparison of three lifting schemes through numerical simulation, addressing the issue of excessive height difference at the matching surface during the lifting process of the steel box girder. Regarding the linearity of bridges, numerous researchers have examined the linear control of steel box girders from various perspectives, including manufacturing and installation processes. Chen [20] and Mei [21] conducted simulation calculations of the unstressed manufacturing geometric shapes, large block installation geometric shapes, bearing pre-offsetting amounts and the girder end rotation angles at the time of temporary matching of the large block steel box girder. Through the whole process control of the construction of the large block steel box girder, the implementation of bridge line shape control has produced positive results. Wang [22] formulated the state transfer equations that describe the transitions among different geometric states of steel box girders by identifying the control parameters associated with these states, and derived the relationships governing geometric state changes during the lifting construction of steel box girders, thereby providing valuable guidance for on-site construction practices. Zhao [23] proposed a method for curvature compensation based on the unstressed geometric characteristics of steel box girders, derived three practical formulas for calculating the curvature compensation angle, and provided a theoretical assessment of the errors associated with the adjusted line shape calculation. Wang [24] examined the influence of various vertical temperature gradient modes on the girder-end pinch angle of welded joints during the erection of large-section steel box girders, proposed a correction method, and analyzed the optimal timing for welding steel box girders to enhance bridge construction practices. Deng [25] explored the impact of temperature gradients on the linear shape during the fabrication stage of large-section steel box girders, evaluated the effects of temperature on the welding process and proposed countermeasures to mitigate temperature-related issues during the factory fabrication of steel box girders. The majority of research on large space structures focused on mechanical properties during integral lifting, while most research on steel box girder structures emphasized the control of bridge alignment and key construction technologies. There was a lack of research on mechanical properties of steel box girder under the condition of lifting point failure in synchronous lifting conditions and displacement error limit under asynchronized lifting conditions. Furthermore, a significant concern was the high coupling stiffness at the lifting point throughout the integral lifting process of large-span steel structures.

In this paper, taking the integral lifting project of the steel box girder of Xiaotun Bridge of Fuyi Expressway as a background, a new integral lifting system and control method were proposed based on the original hydraulic lifting system. The mechanical characteristics of steel box girders subjected to both synchronous and asynchronous integral lifting was analyzed through simulation. Additionally, the extreme scenario of lifting sling failure during synchronous lifting was investigated, and relevant construction control measures were proposed. Ultimately, the mechanical properties of the steel box girder under lifting conditions were analyzed in conjunction with on-site monitoring data.

## 2 Project overview

### 2.1 Project background

**Fig 1:**
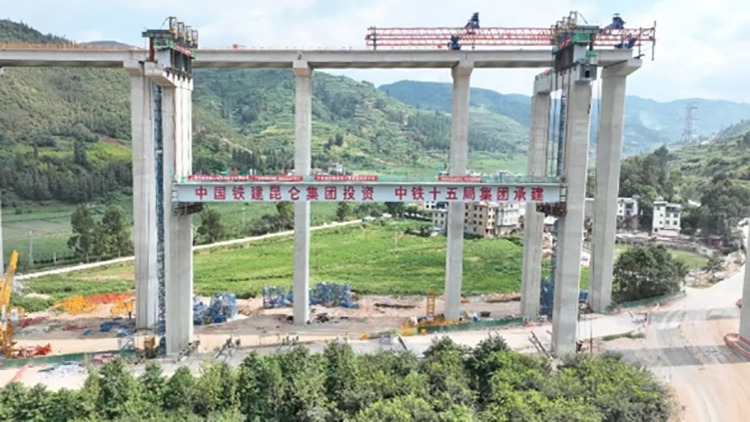
Integral lifting of steel box girder on-site.

Fuyi Expressway is a key project of the “all-access” and “interconnection” project of county expressway in Yunnan Province, which plays a significant role in alleviating traffic congestion in the main city of Kunming and Kunshi Expressway, opening up the economic corridor in the direction of southeast Yunnan, and driving the economic development along Kunming. Among them, the integral lifting construction of the steel box girder for the 6th link of the Xiaotun Bridge is a key project that presents significant challenges. The steel box girder, with a span of 82 m, a total weight of about 920 t and a lifting height of 60.15 m, was installed using the ‘Intelligent Hydraulic Synchronized Lifting Method’. It is the largest span, height and weight of steel box girder installed by this method for a single highway in Yunnan Province at present (Fig 1).

**Fig 2:**
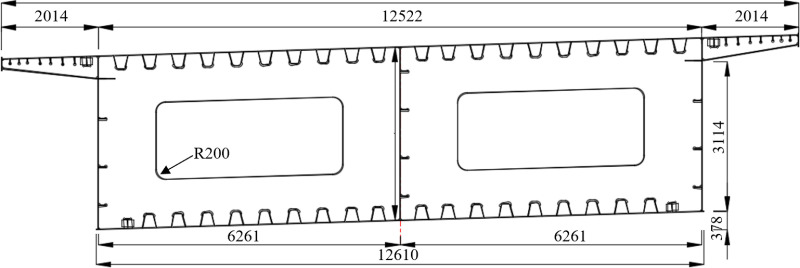
Cross-section of steel box girder (unit: mm).

**Fig 3:**
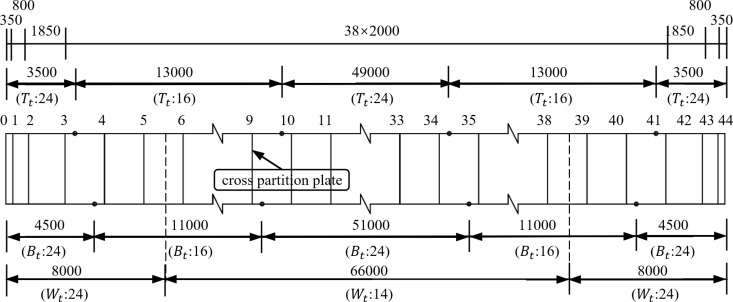
Elevation of steel box girder (unit: mm). ($T_{t}$: the thickness of top plate. $B_{t}$: the thickness of bottom plate. ${\;W}_{t}$: the thickness of web plate.).

The cross-section of steel box girder is single box and double chamber with straight web section, the height of girder is 3.62 m, the width of top plate is 16.55 m, the width of bottom plate is 12.61 m, the length of cantilever is 2.014 m. The top plate and bottom plate each have thickness of 16 mm or 24 mm, the thickness of web plate is 14 mm or 24 mm, and the thickness of cross partition plate at the end fulcrum is 24 mm, the thickness of cross partition plate at other positions is 12 mm. The cross-section and elevation of steel box girder are shown in Fig 2 and Fig 3.

**Fig 4:**
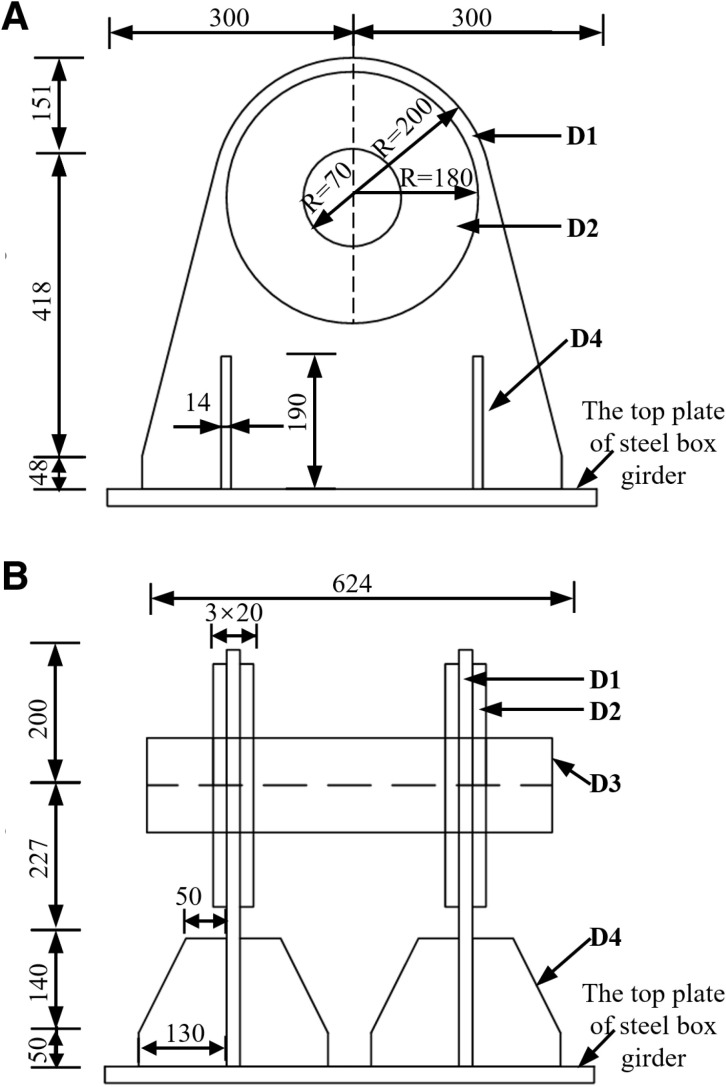
The dimension of lifting lug (unit: mm). (a) Front view. (b) Left view.

Before the integral intelligent lifting of the steel box girder, the lifting lugs were welded to the top plate above the cross partitions of the steel box girder. The ear plate (D1) is a combination of the top disc and the bottom plate trapezoid, with a thickness of 20 mm. The reinforcing plate (D2) is a disc with a radius of 180 mm and a thickness of 20 mm. The pin shaft (D3) is a cylinder with a half diameter of 70 mm and a length of 600 mm. The stiffening plate (D4) is a trapezoid plate with a height of 190 mm and a thickness of 14 mm. The dimensions of the lifting lug is shown in the Fig 4.

### 2.2 Construction challenges

The integral lifting construction of the steel box girder for the Xiaotun Bridge project on the Fuyi Expressway presents the following significant challenges: (1) significant volume of work (lifting section steel box girder span up to 77.22 m, weight up to 866 t, lifting height up to 60.15 m); (2) The lifting process is challenging due to the complex construction conditions, which is constrained by secondary road and the mountainous terrain. The steel box girder project is located in a mountainous region, where the lifting procedure is vulnerable to the effects of sudden wind loads. (3) It is nearly impossible to achieve a completely synchronous lifting state during the lifting process. The steel box girder is bound to exist in an asynchronous lifting state, and it is challenging to determine the warning value of the displacement difference in the asynchronous lifting. (4) The accuracy requirements for installation are exacting. In order to guarantee the reasonableness of the bridge linearity, it is essential to have sufficient precision to ensure that the lifting section and the pier top section of the steel box girder structure closure smoothly.

## 3 Intelligent hydraulic synchronized lifting method

### 3.1 Intelligent lifting system

**Fig 5:**
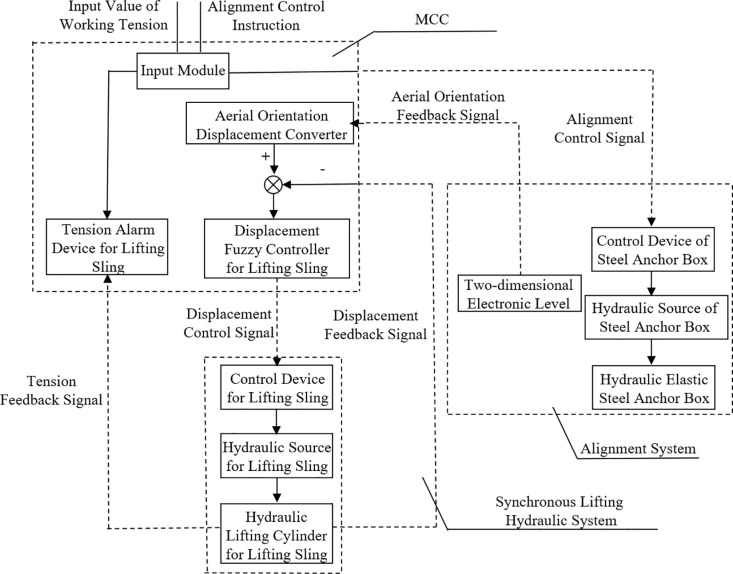
Intelligent hydraulic synchronized lifting system control strategy.

When using intelligent hydraulic synchronized lifting method, to address the challenges related to the steel box girder’s aerial orientation and the regulation of the internal forces, an alignment system was integrated into the existing synchronous hydraulic lifting system. In the process of intelligent hydraulic synchronous lifting of heavy steel box girder, the synchronous lifting hydraulic system and alignment system were connected with the main control computer through wireless communication and controlled by the main control computer in a unified way. The synchronous lifting hydraulic system operates according to the control strategy of “displacement synchronization” and “load balancing”, which is responsible for lifting the steel box girder to the design elevation through lifting slings. The alignment system applies eccentric loading forces to the heavy steel box girder through hydraulic elastic steel anchor box, driving horizontal displacement or rotation of the girder to achieve automatic alignment. The intelligent hydraulic synchronized lifting system control strategy lifting is shown in Fig 5.

The main control computer is equipped with lifting sling tension alarm device, lifting sling displacement fuzzy controller and aerial orientation displacement converter: when the heavy steel box girder is lifted in situ synchronously, the displacement fuzzy controller and the tension alarm are respectively used for feedback control of lifting sling displacement and alarm when the lifting sling tension exceeds the preset threshold, and the aerial orientation displacement converter converts the steel box girder aerial orientation feedback signal into the lifting sling displacement control input signal.

The synchronous lifting hydraulic system comprises control device, hydraulic source and hydraulic lifting cylinder for lifting sling. The control device converts the received lifting sling displacement control signal sent by the main control computer into the control signal of hydraulic source. The hydraulic source executes the control signal of the control device, and pumps the hydraulic oil to the hydraulic lifting cylinder. The hydraulic lifting cylinder drives the lifting sling to move upward, completing the synchronous lifting of the heavy steel box girder and maintaining the aerial orientation when the heavy steel box girder is automatically aligned.

The alignment system includes the control device of steel anchor box, hydraulic source of steel anchor box, hydraulic elastic steel anchor box, and the two-dimensional electronic level. In the process of automatic alignment of heavy steel box girder, the control device converts the received alignment control command into the alignment control signal of the hydraulic source. The hydraulic source executes the control signal of the control device, and pumps the hydraulic oil to the hydraulic elastic steel anchor box. The hydraulic elastic steel anchor box drives the lifting sling to deflect around the hinge joint, applies eccentric load force to the heavy steel box girder, and drives the heavy steel box girder to move horizontally or rotate, so as to realize automatic alignment. At the same time, the two-dimensional electronic level instrument detects the aerial orientation feedback signal in the process of automatic alignment of heavy steel box girder, and transmits it to the main control computer through wireless communication. The main control computer converts it into the lifting sling displacement control signal and transmits it to the lifting sling control device. The lifting sling control device will receive the lifting sling displacement control signal sent by the main control computer and convert it into the control signal of the lifting sling hydraulic source. The lifting sling hydraulic source executes the control signal of the lifting sling control device and pumps the hydraulic oil to the lifting sling hydraulic lifting cylinder, which drives the lifting sling to fall and maintains the aerial orientation of heavy steel box girder during automatic alignment.

### 3.2 Lifting operation of steel box girder

**Fig 6:**
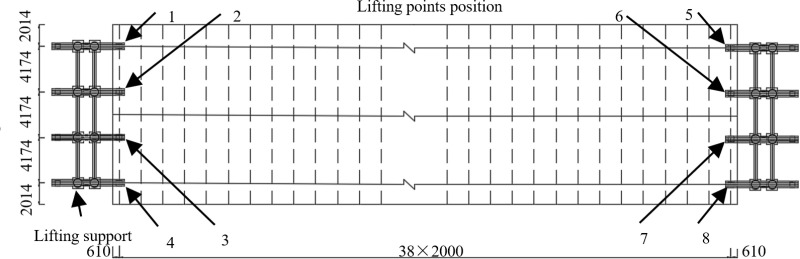
Layout of lifting points of steel box girder (unit: mm).

**Fig 7:**
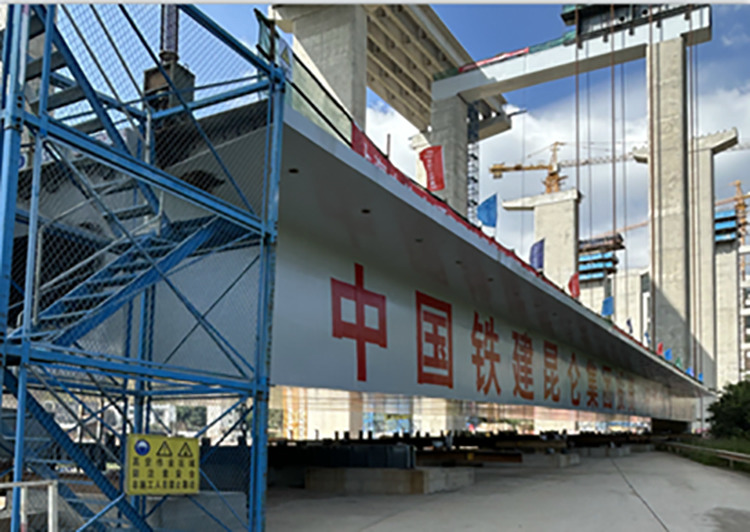
Off-ground inspection of steel box girder.

Before lifting, the weight of steel box girder, temporary structure weight, sling system was carefully calculated, and the possible wind load in the mountainous region was taken into account to ensure that the lifting weight of each lifting point did not exceed 80% of the rated lifting weight [26]. The layout of lifting points of steel box girder is as shown in Fig 6. During the informal lifting operation of the steel box girder, the requisite reach pressure of the hydraulic lifter (factoring in pressure losses) was established on the foundation of the reaction force values at each lifting point. The extension cylinder pressure of the hydraulic lifting system at each lifting point was increased in a graded manner in accordance with the design pressure. Upon the imminent departure of the lifting unit of the steel box girder from the assemble molding frame, it is imperative to reduce the lifting speed and to meticulously monitor the status of each point off the ground. And if necessary do the ‘single-point motion’ lifting technique to ensure the lifting unit is stable. Once the lifting unit has departed from the assemble molding frame by approximately 150 mm, the hydraulic lifting system should be employed to secure the equipment and maintain its position for a period of 12 hours, during which a comprehensive inspection can be conducted, off-ground inspection of steel box girder as shown in Fig 7.

The formal lifting control steel box girder lifting unit is capable of a mean rate of lifting of 2 m/h. For every 10 meters of lifting, measurements were taken at the lifting point. According to the measurement data on the lifting unit aerial orientation fine-tuning (in the fine-tuning before the start of the computer synchronization control system from the automatic mode to switch to manual mode). In the process of integral intelligent lifting on-site, the potential failure of the lifting system may make the asynchronized lifting value of the steel box girder that exceeds acceptable limits, or even make the lifting point fail. This could lead to a change in the lifting orientation of the steel box girder and the internal force exerted by the lifting sling, which could have a significant impact on the safety of the lifting operation. Therefore, it is necessary to study the failure state of the lifting sling of the steel box girder caused by the failure of the lifting system during the lifting process, and to analyze the asynchronous lifting state of the steel box girder.

## 4 Numerical simulation

### 4.1 Numerical model

**Fig 8:**
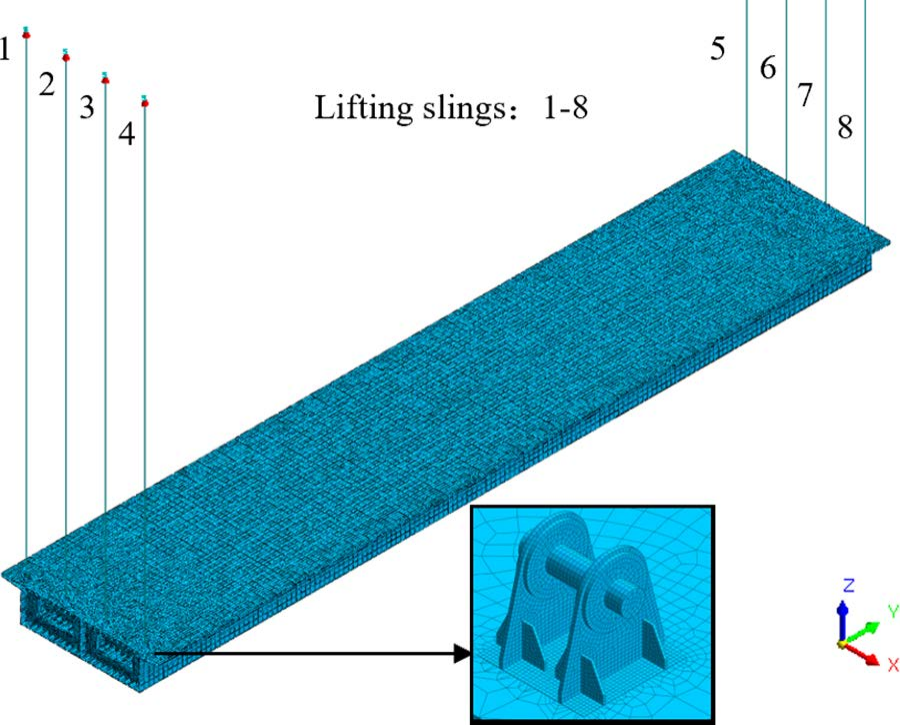
The finite element model of the steel box girder.

Although the integral lifting of the steel box girder is a dynamic process, the acceleration in the lifting process can be ignored because the lifting speed is quite slow, so static analysis is still adopted for the lifting analysis. The fine model of the 77.22 m large-section steel box girder was constructed by the finite element software Midas FEA. The top and bottom plates, stiffeners, transverse partitions, and web plates of the steel box girder were simulated using 3D elements with a mesh size of approximately 500 mm. To improve the accuracy of lifting lug analysis, 3D elements were used with a mesh size of approximately 20 mm. In practical engineering, the bottom plate of the lifting lug was welded and fixed to the top plate of the steel box girder, and geometric connections were made before mesh dividing in the model. The lifting sling adopts 1D tension only element simulation, which can ensure the coupling between the lifting sling and the lifting lug node. The finite element model consists of 406098 nodes and 910615 elements, as shown in Fig 8.

The steel box girder and lugs were constructed using Q345qC and Q345qD steel, respectively, and lifting sling were constructed using Strand1860. According to the Technical Code for Integral Lifting of Heavy Structure and Equipment(GB 51162−2016) [26] and Structural Steel for Bridges (GB/T714-2015) [27], the thickness of each steel component is less than 50 mm, with a yield strength of 345 MPa. The elasticity modulus is 206 GPa, Poisson’s ratio is 0.31, and the bulk density is 78.5 kN/m^3^. The tensile strength of the lifting sling is 1860 MPa, the elastic modulus is 195 GPa, the Poisson’s ratio is 0.3, and the minimum breaking load is 355 kN.

When analyzing the lifting process of steel box girders, the load combination was selected according to code [26], and the calculated load considers the weight of the components and the horizontal wind load (0.53 kN/m^2^). Since the steel box girder was only subjected to vertical constraints of the sling during the integral lifting process, the model boundary condition was to apply the vertical displacement constraints at the positions of each lifting point, and to apply the horizontal elastic constraints in view of the insufficient constraints of the model.

### 4.2 Lifting conditions

The most unfavorable situation in the integral lifting process of the steel box girder is the failure of the lifting sling, which may cause the steel box girder to be partially damaged or to fall suddenly during the lifting process. To prevent related accidents, various lifting sling failure conditions (Table 1) were analyzed. Condition 1 represents the normal lifting condition of the steel box girder, while conditions 2–8 depict extreme lifting conditions.

**Table 1:** Integral lifting conditions.

Working conditions	Number of failed slings	Lifting sling position	Failure category
**1**	0	–	–
**2**	1	1	Single side lifting sling
**3**	1	2	Single central lifting sling
**4**	2	2,3	Two central lifting slings at the same end
**5**	2	2,6	Two central lifting slings on opposite at both ends
**6**	2	2,7	Two central lifting slings with staggered ends
**7**	2	1,5	Two side lifting slings on opposite at both ends
**8**	2	1,8	Two side lifting slings with staggered ends

## 5 Results and discussion

### 5.1 Research on synchronous lifting

**Fig 9:**
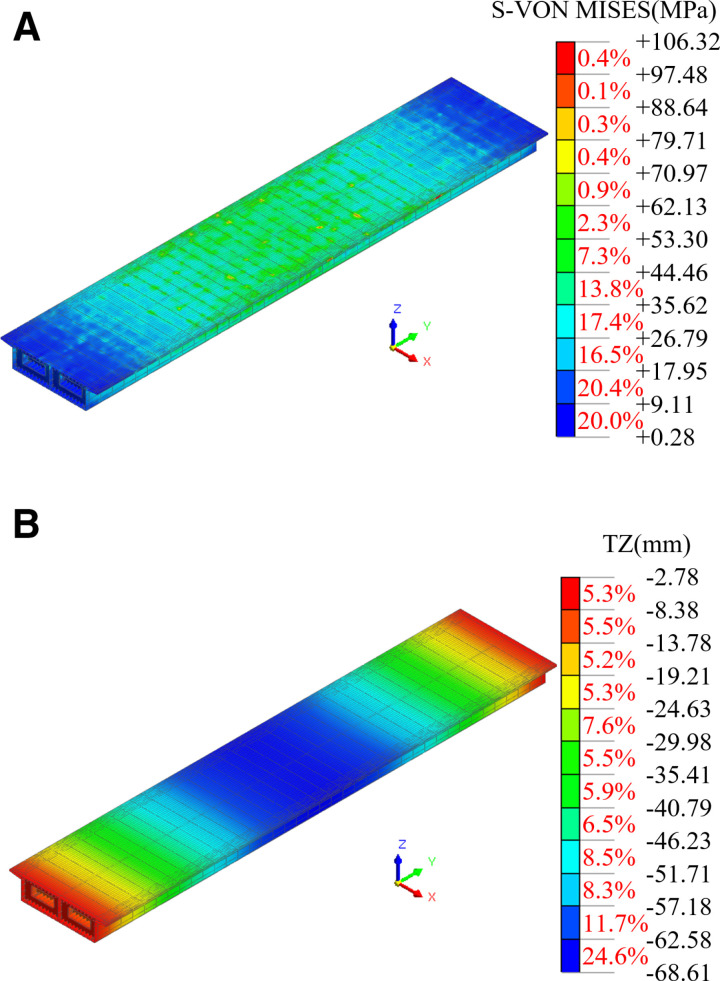
Mechanical properties of steel box girders under normal lifting conditions. (a) S-von Mises. (b) Vertical displacement.

**Fig 10:**
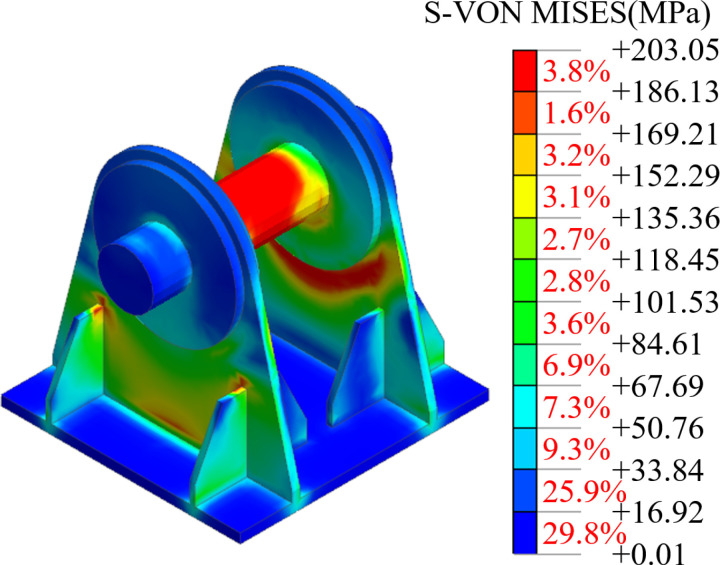
The stress of the lifting lugs under normal lifting conditions.

The lifting force at each lifting point, as well as the stress and deformation of the steel box girder, were analyzed under various working conditions. Among them, the distributions of stresses and vertical displacements of the steel box girder, and the distributions of lug stresses, under Working Condition 1 were illustrated in Figs 9 and 10. As shown in the Figs, the steel box girder exhibits deformation characteristics typical of a simply supported girder under normal lifting conditions. The stress and vertical displacement of the steel box girder gradually increase from both ends toward the center of the span, with a maximum vertical displacement of 68.61 mm. This displacement is less than 77220 mm/500 = 154.44 mm, which complies with the Specifications for Design of Highway Steel Bridge (JTG D64-2015) [28]. During the lifting process, the steel strand was positioned at the center of the pin, where the maximum stress of 203.05 MPa occurred. The stress at other locations on the lugs was relatively low, and the structural strength of the lugs met the requirements outlined in the code [27].

The results of the lifting force calculations for the lifting sling under each working condition are presented in Table 2. These results are categorized into four groups based on failure types: Working Condition 1 represents the normal lifting state of the steel box girder, while Working Conditions 2 and 3 correspond to the failure states of a single lifting sling of the steel box girder. Working Conditions 4, 5, and 6 depict the failure states of two central lifting slings of the steel box girder, and Working Conditions 7 and 8 illustrate the failure states of two side lifting slings of the steel box girder. The simulation results for Working Condition 1 serve as a control for comparing the variations in the lifting force values, as shown in the Table 2.

**Table 2:** Lifting force at each lifting point under different working conditions.

Lifting point	Lifting force under different working condition (kN)							
	1	2	3	4	5	6	7	8
**1**	1065.31	–	1559.25	2135.18	1567.66	1546.51	–	–
**2**	1073.94	1968.44	–	–	–	–	2114.17	1950.32
**3**	1070.39	1365.17	1544.78	–	1541.17	1537.82	1419.80	1358.00
**4**	1071.56	951.63	1181.33	2150.14	1172.51	1200.97	751.29	977.03
**5**	1069.72	1084.08	1106.62	1069.89	1570.67	1210.98	–	948.79
**6**	1067.25	1078.38	1084.64	1067.31	–	1523.15	2096.52	1311.57
**7**	1072.82	1062.98	1055.19	1072.76	1556.70	–	1423.64	2024.99
**8**	1071.41	1059.81	1038.73	1071.24	1157.86	1551.13	765.13	–

(1) In the case of a single sling failure, only the lifting force of the sling adjacent to the failed sling changes significantly. When lifting sling 1 failed, the lifting force at the adjacent lifting sling 2 increased from 1073.94 kN to 1968.44 kN, representing a rise of 83.29%. When lifting sling 2 failed, the lifting forces at adjacent lifting slings 1 and 3 increased from 1065.31 kN and 1070.39 kN to 1559.25 kN and 1544.78 kN, respectively, reflecting increases of 46.37% and 44.32%. The failure of side lifting sling had a greater impact on the internal force changes of adjacent lifting slings than the failure of central lifting sling.
(2) In the two lifting slings at the central of the steel box girder simultaneous failure states, when lifting slings 2 and 3 failed simultaneously, the lifting force of lifting slings 1 and 4 increases by 100.43% and 100.66%, respectively. When the failed sling was located at both ends of the steel box girder, the maximum lifting force of the sling is 1570.67 kN, an increase of 46.83%. It was crucial to consider the conditions under which two central lifting slings fail at the same end of the girder.
(3) In the two lifting slings at the edge of the steel box girder simultaneous failure states, with the central lifting slings became the primary load-bearing slings. When lifting slings 1 and 5 failed simultaneously, the steel box girder exhibited a tendency of overturn, the lifting forces at lifting slings 2 and 6 increased dramatically by 96.86% and 96.44%, respectively. When lifting slings 1 and 8 failed at the same time, the steel box girder exhibited a tendency of torsion, the lifting force of the central lifting slings 2 and 7 increases by 81.60% and 88.75%, respectively.

The content presented above indicates that the failure of the lifting point during the lifting process will result in a redistribution of the internal forces within the lifting sling. The lifting sling consists of 15*ϕ*17.80 mm steel strands, with the breaking force of a single strand being no less than 355 kN. To ensure the safety of the steel box girder lifting sling under extreme lifting conditions, the safety coefficient was calculated as follows: 355 ÷ (lifting force at the lifting point/number of strands). The safety coefficients of the steel strands for conditions 1–8 were 4.96, 2.71, 3.42, 2.48, 3.39, 3.43, 2.52, and 2.63, respectively. Although the failure of the lifting point reduces the safety coefficient of the lifting sling, it still meets the required safety standards.

**Fig 11:**
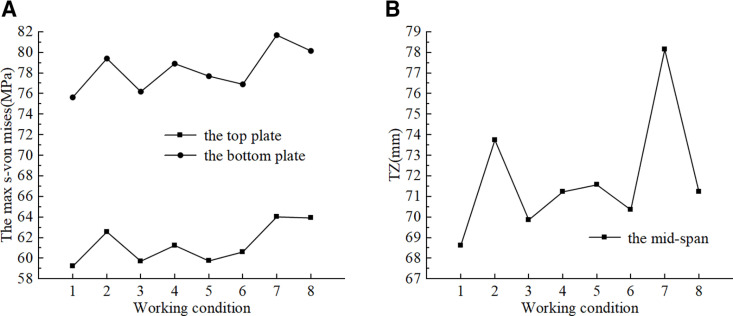
Mechanical properties of steel box girders under different working conditions. (a) S-von Mises. (b) Vertical displacement.

The results of the mechanical property calculations for the steel box girder are presented in Fig 11. Under different working conditions, the steel box girder exhibited force-deformation characteristics similar to those of simply supported beams. The extreme state of steel box girder lifting sling failure was compared to the normal lifting state, there was no significant variation in the mid-span deflection or the stresses in the top and bottom plates of the steel box girder. The maximum von Mises stresses in the top and bottom plates, as well as the mid-span vertical displacement, increased within the ranges of 0.8% to 8.1%, 0.7% to 8.0%, and 1.8% to 13.9%. The strength and deformation of the steel box girder met the codes [27,28]. However, it is essential to consider the impact of lifting deformation on the linearity of the bridge during construction and to establish a reasonable pre-bend based on the simulated values.

**Fig 12:**
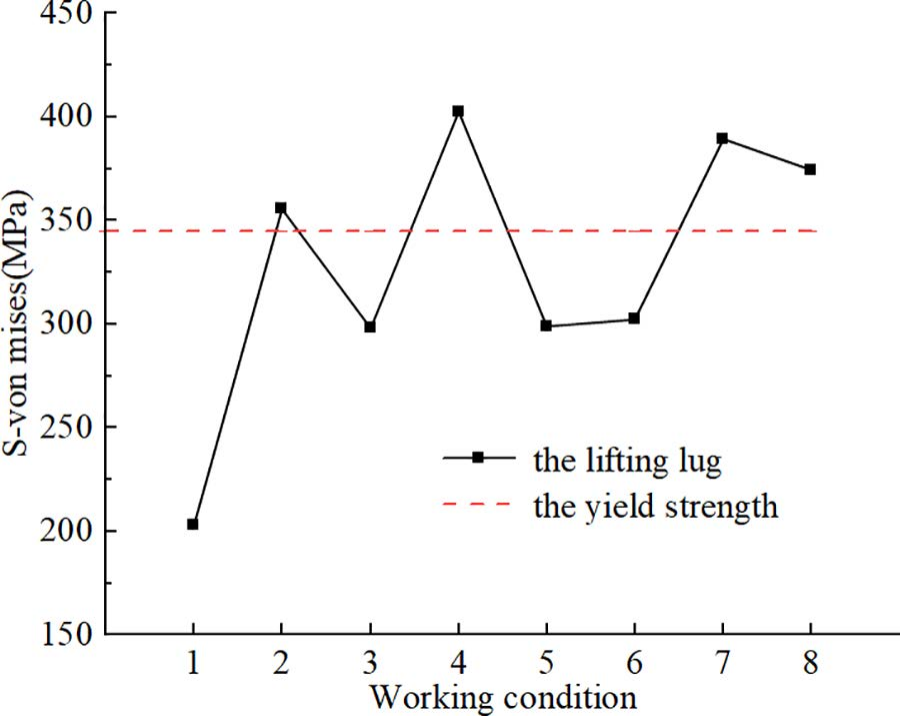
The maximum von Mises stress on the lifting lug.

The maximum von Mises stress in the lifting lugs was calculated as illustrated in Fig 12. When the single lifting sling failed, the lifting force at lifting point 2 in working condition 2 increased, resulting in a maximum von Mises stress of 355.29 MPa in the lifting lugs. When the two central lifting slings failed, a significant safety hazard arose in working condition 4, as the lifting lug stress at the side lifting point on the same end reached 402.33 MPa. When the two lifting slings at the edge failed, the stress on the lugs exceeded the yield stress. It was crucial to monitor the results from the intelligent lifting system regarding the lifting slings and lugs at the edge throughout the lifting process, and appropriate measures should be implemented to reinforce the lugs.

### 5.2 Research on asynchronous lifting

With respect to the substantial volume of the integral lifting project involving the steel box girder, it is essential to calculate the asynchronous effects at each lifting point [26]. The asynchronous lifting in the model was achieved through displacement method, which applied forced displacement to the lifting slings to achieve rigid body displacement caused by asynchronous lifting of the steel box girder. As illustrated in Fig 12 the failure of lifting slings under working conditions 2, 4, 7, and 8 would lead to yield of the lifting lugs, which would significantly adversely affect the lifting of the steel box girder. Therefore, these slings were utilized as a reference for establishing the asynchronous lifting condition. The asynchronous lifting sling setting as shown in Table 3.

**Table 3:** Asynchronous lifting sling setting.

Lifting condition	Lifting sling setting
**I**	Lifting sling 1 is forced to move 15 mm forward along the Z axis
**II**	Lifting slings 2,3 is forced to move 15 mm forward along the Z axis
**III**	Lifting slings 1,5 is forced to move 15 mm forward along the Z axis
**IV**	Lifting slings 1,8 is forced to move 15 mm forward along the Z axis

**Fig 13:**
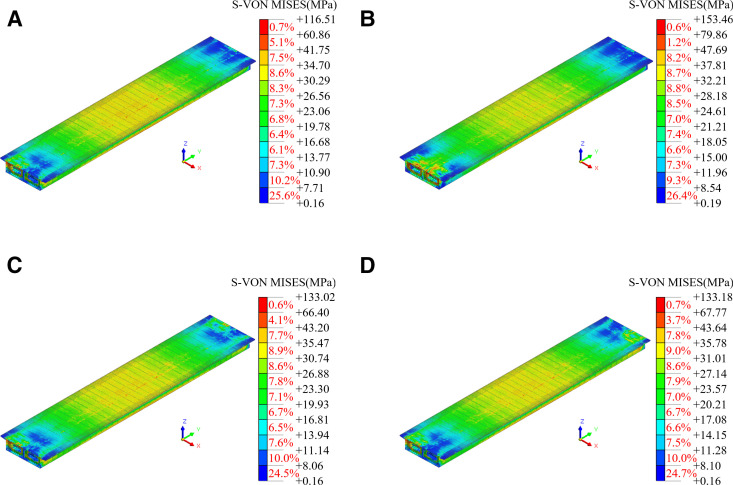
The stress cloud for each lifting condition. (a) I. (b) II. (c) III. (d) IV.

**Fig 14:**
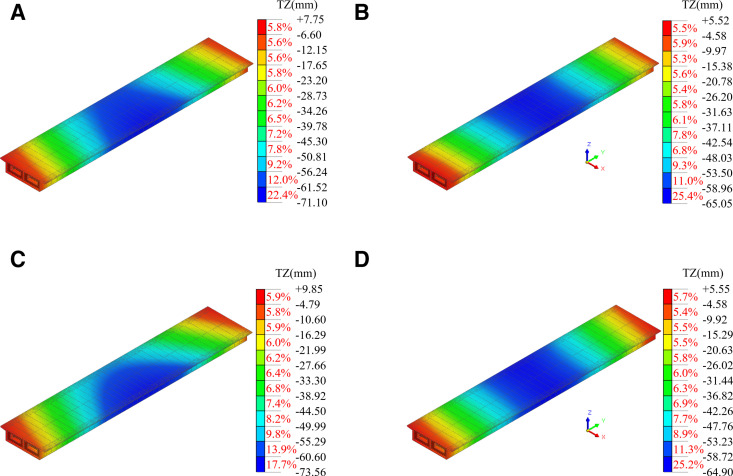
The displacement cloud for each lifting condition. (a) I. (b) II. (c) III. (d) IV.

Finite element analysis was conducted for the different lifting conditions, and the stress and vertical displacement cloud of the steel box girder under these conditions are presented in Figs 13 and 14. As illustrated in Fig 13, due to the forced displacement applied to the lifting sling, the maximum von Mises stress occurs near the lifting sling, while the displacement of the steel box girder caused by asynchronous lifting was a rigid body displacement, which had a relatively small impact on the stress changes of the steel box girder. The lifting sling in lifting condition II was the closest, resulting in a maximum stress value of 153.46 MPa at this location of steel box girder. The stress results for all four conditions met the code [27]. The stress distribution along the box girder at other locations mirrored that of the normal lifting condition, with stress values gradually increasing from the ends of the box girder toward the center of the span. But to ensure the safety of the lifting, it should be ensured that the lifting slings were located above the transverse partition of the steel box girder, and the transverse partition and panel positions inside the girder should be reinforced by adding welded steel plates.

As shown in Fig 14, due to the high overall stiffness of the steel box girder, asynchronous lifting would cause its rigid body displacement, resulting in a change in the transverse slope of the bridge. In working condition II, the vertical displacement cloud map of the steel box girder was similar to that of the normal lifting state, indicating that when the double lifting slings were located at the same end, the impact on the vertical displacement and transverse slope of the steel box girder was relatively small. The steel box girder exhibited tendencies of overturn or torsion in conditions III and IV, respectively. However, considering the large size of the steel box girder structure and the only 0.1% change in the transverse slope at both ends, the effect of asynchronous lifting was relatively small, and the calculation results met the code [28].

Combined with Figs 13 and 14, it was evident that, under the asynchronous lifting of the steel box girder within the specified error limits, both the stress and deformation of the steel box girder met the codes [27,28].Although the probability of encountering extreme lifting conditions for the steel box girder is minimal, it remains essential to monitor key components to ensure construction safety.

### 5.3 Mechanical properties of lifting support

**Fig 15:**
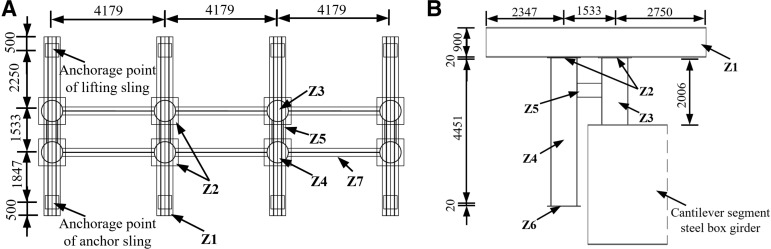
The dimensions of the lifting support (unit: mm). (a) Plan view. (b) Left view.

According to the construction requirements, the project must install a lifting support at the top of the side pier and position lifting equipment on the lifting support to facilitate the complete intelligent lifting operation of the steel box girder. When the lifting support was installed on site, it was assembled as a whole on the ground and lifted to the corresponding position of the upper lifting point by truck crane. The outer diameter of the column (Z3 and Z4) is 800 mm and the wall thickness is 16 mm; The outer diameter of the connecting cylinder (Z5) is 426 mm and the wall thickness is 8 mm; The side length of the cushion block (Z2 and Z6) is 1000 mm and the thickness is 20 mm; The section size of connecting H-shaped steel (Z7) is HN700 × 300 × 13 × 24 mm, and the lifting girder (Z1) is double jointed HN900 × 300 × 16 × 28 mm steel. Among them, Q235 steel is used for column (Z3 and Z4), connecting cylinder (Z5), cushion block (Z2 and Z6) and connecting H-shaped steel (Z7), while Q355 steel is used for lifting girder (Z1). The dimensions of the lifting support are shown in Fig 15.

**Fig 16:**
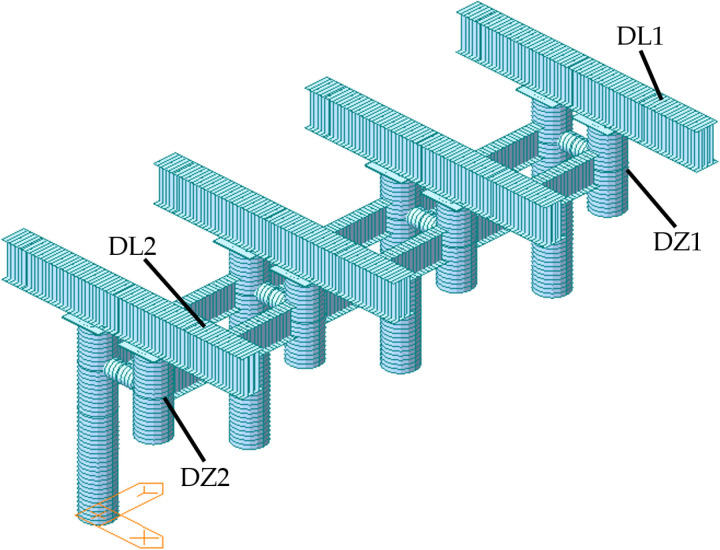
Layout of strain measurement points of lifting support.

The lifting support structure is a critical temporary component in the integral intelligent lifting process of steel box girders, and its safety was paramount. Therefore, this paper utilized finite element software to analyze the mechanical characteristics of the lifting support. The load combination was selected according to the Technical Code for Integral Lifting of Heavy Structure and Equipment(GB 51162−2016) [26]. The load parameters were taken according to the design, taking into account the weight, lifting force (1038 kN), temperature load (±25°C) and wind load (0.87 kN/m^2^). The front lifting column (Z3) is circumferentially welded to the cantilevered segment of the pier-top steel box girder, while the rear lifting column (Z4) is connected to embedded bearing blocks (Z6) through pre-installed anchor bolts. Consequently, the boundary conditions for the finite element model of the lifting frame are defined as fixed constraints at the base of both front and rear lifting columns. The finite element model of the lifting support has 866 nodes and 864 units, the finite element model of lifting support and the layout of strain measuring points are shown in Fig 16.

**Fig 17:**
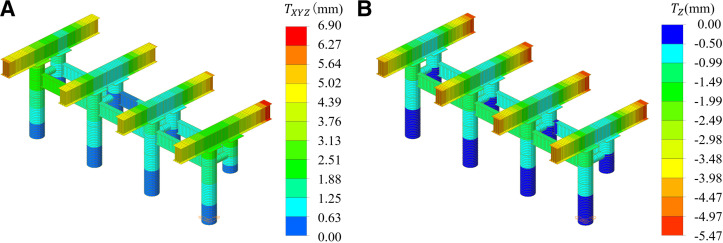
The displacement of the lifting support. (a) Overall displacement. (b) Vertical displacement.

During the integral intelligent lifting of the steel box girder, the upper edge of the lifting girder experienced tension, while the lower edge underwent compression. The stress levels changed from −211.40 MPa to 211.40 MPa, with the maximum stress occurring above the front column. The column itself was subjected to compression, with stress values changed from −97.71 MPa to −8.76 MPa, and the peak stress was located at the top of the column. The simulated stress values at the measuring points of the lifting support are presented in Table 4. The total maximum displacement of the lifting support is 6.90 mm, and the maximum vertical displacement is 5.47 mm. The displacement of the lifting support is shown in Fig 17. Additionally, the first-order safety coefficient of the column structure was calculated to be 24.40, indicating that the mechanical properties of the column structure met the code for Specifications for Design of Highway Steel Bridge (JTG D64-2015) [28].

**Table 4:** The simulated stress of lifting support.

Monitoring point	DL1	DL2	DZ1	DZ2
**Stress(MPa)**	45.13	45.22	−71.58	−71.47

### 5.4 Discussion

During the lifting process, the steel box girder exhibits the characteristics of a simply supported beam. When the lifting system malfunctions and the lifting sling fails, it can cause the internal force of the lifting sling to redistribute, resulting in a decrease in the safety factor of the lifting sling and possibly causing the lifting lugs to yield. The closer the distance to the failed sling, the greater the change in lifting force. The failure of the suspension cable has a significant impact on the safety factor of adjacent lifting slings and the stress of the lifting lugs, while it has a relatively small impact on the mid span stress and displacement of the steel box girder.

Due to the high overall stiffness of the steel box girder, when the steel box girder is lifted asynchronously, rigid body displacement will occur, resulting in a change in the transverse slope of the girder. At this time, attention should be paid to the value of the displacement difference in the asynchronous lifting. When the displacement difference is large, it will lead to the overturn or torsion of the steel box girder. The asynchronous lifting value within the allowable error range has a small impact on the stress and displacement changes of the steel box girder, but the girder section below the lifting lugs should be reinforced.

## 6 Engineering applications

### 6.1 Design of monitoring scheme

**Fig 18:**
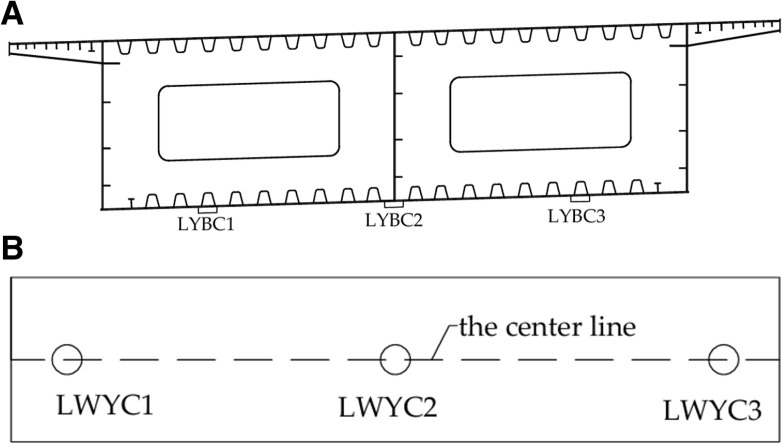
Layout of measurement points for steel box girder. (a) Layout of strain measurement points in mid-span section. (b) Layout of displacement measurement points (end of girder, mid-span).

Based on the finite element analysis conducted, it has been determined that the stress, linear shape, and lifting force at the lifting point of the steel box girder are the primary monitoring parameters. Prior to the commencement of the integral synchronous intelligent lifting of the steel box girder, several key settings must be established, including the maximum working pressure of the hydraulic lifter and the maximum allowable stroke difference for each lifting point to remain in synchronism. The simulation results obtained under normal lifting state of the steel box girder will serve as the early warning values for monitoring during the integral lifting stage. The steel box girder was arranged with 6 monitoring points (3 strain measuring points and 3 displacement measuring points), and the arrangement of the measuring points was as shown in Fig 18, and the single lifting support was arranged with a total of 4 monitoring points (the arrangement of 4 strain measuring points was as shown in Fig 16).

Through real-time monitoring of the hydraulic lifter’s working pressure, along with the recording of additional monitoring parameters, the main control computer processes and preserves a variety of monitoring data. If the monitored value exceeds the warning threshold and shows a significant upward trend, the computer will lock all hydraulic lifters and suspend lifting operations until the cause of the anomaly is determined.

### 6.2 Results of monitoring

**Fig 19:**
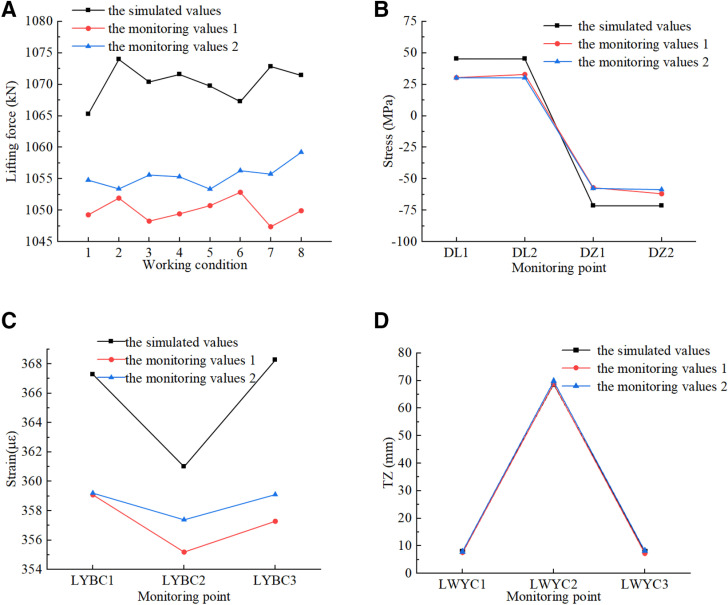
Comparative of each parameter. (a) Lifting point. (b) Lifting support. (c) Steel box girder. (d) Steel box girder.

Prior to the commencement of the integral synchronous intelligent lifting of the steel box girder, measurement points were established in accordance with the monitoring scheme. A wireless stress detection system was employed for real-time monitoring throughout the lifting process. To validate the accuracy of the simulation results, the measured data were compared and analyzed against the numerical simulation. The comparison includes the lifting force at each lifting point, the stress on the lifting support, as well as the strain and displacement within the span of the steel box girder, as illustrated in Fig 19. The following conclusions can be drawn:

(1) For safety reasons, the sudden 8-level wind load at the bridge location was considered in the numerical simulation, resulting in simulated lifting force and lifting support stress being greater than the monitoring values, while the wind load had a relatively small impact on the mid span stress and displacement of the steel box girder. During actual lifting process, when the wind speed reached level 6, lifting would be halted, and appropriate stabilization measures were implemented.
(2) The error range between the simulated values of the lifting force at each lifting sling and the actual monitoring values was between 1.0% and 2.4%, and the actual lifting force was about 1053 kN. The monitoring values of key measurement points for steel box girder and the lifting support were aligned closely with the simulated values. The mid-span stress and displacement of the steel box girder are approximately 74 MPa and 70 mm, both of which met the codes [27,28] and exhibited a relatively high safety margin.
(3) The load and boundary conditions of the numerical simulation were accurate. The results of the lifting force at each lifting point in other extreme lifting states, as well as the mechanical properties of the steel box girder, provided valuable reference for adjustment of actual construction measures.

## 7 Conclusions

Taking the Xiaotun Bridge project of the Fuyi Expressway as the engineering background, this paper discussed the key technologies involved in the integral intelligent lifting of large-span steel box girders. A comprehensive intelligent lifting scheme for these girders was proposed, and the mechanical properties in both synchronous and asynchronous lifting states were analyzed using Midas FEA finite element software. The main conclusions are as follows:

1. In view of the constraints imposed by on-site construction conditions and the requirements of construction progress, a novel construction method was proposed. This method incorporated synchronous hydraulic lifting, intelligent monitoring and control, automatic alignment, and other advanced construction technologies, in addition to informal lifting and micromotion lifting. The automated computer control system enabled the adjustment of the aerial orientation of the steel box girder and regulated the stress values of each component, which ensured the safe lifting of the steel box girder as a cohesive unit.
2. Under synchronous lifting conditions, the steel box girder exhibited stress characteristics similar to those of a simply supported beam, while the mechanical properties of the lifting support met the safety requirements. When a single side lifting sling failed or a single central lifting sling failed, the internal force in the adjacent lifting slings increased by 83.29% and 46.37%, respectively. Furthermore, the maximum internal force in the sling reached 2150.14 kN, representing an increase of 100.66% when both lifting slings failed. The failure of the lifting slings led to a redistribution of internal forces in the lifting slings. Concurrently, the maximum von Mises stress values of the top and bottom plates of the steel box girder, as well as the mid-span vertical displacements under different working conditions, increased within the ranges of 0.8% ~ 8.1%, 0.7% ~ 8.0% and 1.8% ~ 13.9%, respectively.
3. Under asynchronous lifting conditions, the transverse slope of the steel box girder changed, and the maximum stress value increased. The maximum von Mises stress value of 153.46 MPa occurred when the two middle lifting slings at the same end were utilized. The steel box girder exhibited a tendency to overturn or torsion when the two opposite or staggered-side lifting slings at both ends were employed, but the change in the transverse slope of the bridge at both ends was 0.1%; The stress and deformation of the steel box girder met the relevant codes under asynchronous lifting within displacement error limits.
4. The error range between the actual monitoring data and the theoretical data for the lifting force at each lifting point was between 0.1% and 2.4%. The monitoring data for both the steel box girder and the lifting support indicated that they possessed a significant safety factor, ensured the structural integrity. The integral lifting process of the steel box girder successfully achieved both accuracy and safety. Additionally, the simulation results regarding the failure of the lifting point under synchronous lifting conditions of the steel box girder can provide a certain reference for addressing unexpected situations in similar integral lifting projects.
